# Leveraging a foundation model zoo for cell similarity search in oncological microscopy across devices

**DOI:** 10.3389/fonc.2025.1480384

**Published:** 2025-06-18

**Authors:** Gabriel Kalweit, Anusha Klett, Paula Silvestrini, Jens Rahnfeld, Mehdi Naouar, Yannick Vogt, Diana Infante, Rebecca Berger, Jesús Duque-Afonso, Tanja Nicole Hartmann, Marie Follo, Elitsa Bodurova-Spassova, Michael Lübbert, Roland Mertelsmann, Joschka Boedecker, Evelyn Ullrich, Maria Kalweit

**Affiliations:** ^1^ Collaborative Research Institute Intelligent Oncology (CRIION), Freiburg, Germany; ^2^ Neurorobotics Lab, Department of Computer Science, University of Freiburg, Freiburg, Germany; ^3^ Laboratory of Applied Cellular and Molecular Biology, Institute of Veterinary Sciences of the Litoral, Universidad Nacional del Litoral (UNL) - National Scientific and Technical Research Council (CONICET), Esperanza, Argentina; ^4^ Department of Medicine I, Medical Center - University of Freiburg, Faculty of Medicine, University of Freiburg, Freiburg, Germany; ^5^ Lighthouse Core Facility, Medical Center – University of Freiburg, Faculty of Medicine, University of Freiburg, Freiburg, Germany; ^6^ German Cancer Consortium (DKTK) and German Cancer Research Center (DKFZ), Partner Site Freiburg, Freiburg, Germany; ^7^ Mertelsmann Foundation, Freiburg, Germany; ^8^ Intelligent Machine-Brain Interfacing Technology (IMBIT), BrainLinks-BrainTools, University of Freiburg, Freiburg, Germany; ^9^ Department of Pediatrics, Experimental Immunology and Cell Therapy, Goethe University Frankfurt, Frankfurt am Main, Germany; ^10^ German Cancer Consortium (DKTK) and German Cancer Research Center (DKFZ), Partner Site Frankfurt, Frankfurt am Main, Germany

**Keywords:** artificial intelligence, deep learning, foundation models, nearest neighbor search, cell imaging

## Abstract

**Background:**

Cellular imaging analysis using the traditional retrospective approach is extremely time-consuming and labor-intensive. Although AI-based solutions are available, these approaches rely heavily on supervised learning techniques that require high quality, large labeled datasets from the same microscope to be reliable. In addition, primary patient samples are often heterogeneous cell populations and need to be stained to distinguish the cellular subsets. The resulting imaging data is analyzed and labeled manually by experts. Therefore, a method to distinguish cell populations across imaging devices without the need for staining and extensive manual labeling would help immensely to gain real-time insights into cell population dynamics. This especially holds true for recognizing specific cell types and states in response to treatments.

**Objective:**

We aim to develop an unsupervised approach using general vision foundation models trained on diverse and extensive imaging datasets to extract rich visual features for cell-analysis across devices, including both stained and unstained live cells. Our method, Entropy-guided Weighted Combinational FAISS (EWC-FAISS), uses these models purely in an inference-only mode without task-specific retraining on the cellular data. Combining the generated embeddings in an efficient and adaptive k-nearest neighbor search allows for automated, cross device identification of cell types and states, providing a strong basis for AI-assisted cancer therapy.

**Methods:**

We utilized two publicly available datasets. The WBC dataset includes 14,424 images of stained white blood cell samples from patients with acute myeloid and lymphoid leukemia, as well as those without leukemic pathology. The LISC dataset comprises 257 images of white blood cell samples from healthy individuals. We generated four in-house datasets utilizing the JIMT-1 breast cancer cell line, as well as Jurkat and K562 (leukemic cell lines). These datasets were acquired using the Nanolive 3D Cell Explorer-fluo (CX-A) holotomographic microscope and the BioTek Lionheart FX automated brightfield microscope. The images from the in-house datasets were manually annotated using Roboflow software. To generate the embeddings, we used and optimized a concatenated combination of SAM, DINO, ConvNeXT, SWIN, CLIP and ViTMAE. The combined embeddings were used as input for the adaptive k-nearest neighbor search, building an approximate Hierarchical Navigable Small World FAISS index. We compared EWC-FAISS to fully fined-tuned ViT-Classifiers with DINO-, and SWIN-backbones, a ConvNeXT architecture, as well as to NMTune as a lightweight domain-adaptation method with frozen backbone.

**Results:**

EWC-FAISS performed competitively with the baselines on the original datasets in terms of macro accuracy. Macro accuracy is the average of class-specific accuracies, treating all classes equally by averaging their individual accuracies. EWC-FAISS ranked second for the WBC dataset (macro accuracy: 97.6 ± 0.2), first for cell state classification from Nanolive (macro accuracy: 90 ± 0), and performed comparably for cell type classification from Lionheart (macro accuracy: 87 ± 0). For the transfer to out-of-distribution (OOD) datasets, which the model had not seen during training, EWC-FAISS consistently outperformed the other baselines. For the LISC dataset, EWC-FAISS achieved a macro accuracy of 78.5 ± 0.3, compared to DINO FT’s 17 ± 1, SWIN FT’s 44 ± 14, ConvNeXT FT’s 45 ± 9, and NMTune’s 52 ± 10. For the cell state classification from Lionheart, EWC-FAISS had a macro accuracy of 86 ± 1, while DINO FT, SWIN FT, and ConvNeXT FT achieved 65 ± 11, 68 ± 16, and 81 ± 1, respectively, and NMTune 81 ± 7. For the transfer of cell type classification from Nanolive, EWC-FAISS attained a macro accuracy of 85 ± 0, compared to DINO FT’s 24.5 ± 0.9, SWIN FT’s 57 ± 6, ConvNeXT FT’s 54 ± 4, and NMTune’s 63 ± 4. Additionally, building EWC-FAISS after embedding generation was significantly faster than training DINO FT (∼ 6 minutes compared to *>* 10 hours). Lastly, EWC-FAISS performed comparably in distinguishing cancerous cell lines from Peripheral Blood Mononuclear Cells with a mean accuracy of 80 ± 5, compared to CellMixer with a mean accuracy of 79.7.

**Conclusion:**

We present a novel approach to identify various cell lines and primary cells based on their identity and state using images acquired across various imaging platforms which vary in resolution, magnification and image quality. Despite these differences, we could show that our efficient, adaptive k-nearest neighbor search pipeline can be applied on a large image dataset containing different cell types and effectively differentiate between the cells and their states such as live, apoptotic or necrotic. There are several applications, particularly in distinguishing various cell populations in patient samples or monitoring therapy.

## Highlights

- Foundation models, even without domain-specific training, provide effective discriminative features for both stained and unstained cellular imaging. EWC-FAISS as an adaptive k-nearest neighbor search on embeddings generated from a combination of foundation models achieved high accuracy across various datasets.
- General features from a combination of foundation models demonstrated superior transferability to new experimental settings, including both stained and unstained cells across different recording devices.
- Fast inference with EWC-FAISS allows for quick development cycles, supporting efficient differentiation of cell types and states, crucial for AI-assisted cancer therapy.
- A combination of DINO, ConvNeXT and SWIN proved to be a general, robust and versatile combination leading to good transfer performances in cell state and type classification.

## 1 Introduction

In medical and biological research, data acquisition is often challenging and involves high costs and labor-intensive processes (1). This is especially true in cellular imaging analysis, where traditional approaches rely heavily on supervised learning techniques that require high-quality, large-scale labeled datasets, which are expensive and time-consuming to produce. Additionally, the heterogeneity of imaging equipment (e.g., different microscopes) and protocols (e.g., varying media and lighting conditions) introduces variability, complicating the task and degrading the performance of narrowly trained models. Training these models demands costly GPUs, extensive training time, and frequent retraining for new tasks. Addressing these challenges necessitates methodologies that leverage existing data more efficiently and generalize across diverse imaging conditions without extensive retraining or fine-tuning.

Recent advancements in machine learning, particularly in the development of *general* foundation models (2), present a promising solution. Particularly models like DINO (3, 4) and Segment Anything (SAM) (5), have significantly influenced medical and cellular image processing domains. MedSAM (6) has extended the utility of SAM to general medical imaging tasks, while models like UNI (7), WTC-11 DINO (8), DINOBloom (9) and scDINO (10) have adapted DINO-style approaches to histopathology and (multi-channel) cellular image analysis. The scDINO (10) model demonstrated that a k-nearest neighbor (k-NN) search using DINO features, fine-tuned and adapted to multi-channel cellular imaging, can be competitive with other methods for cell classification tasks. Israel et al. (11) introduced with CellSAM an adaptation of SAM specifically designed for cell segmentation. Also, self-supervised masked autoencoders have been shown to be capable of capturing cellular biology when trained on massive datasets (12). Despite these advancements, training foundation models specifically for medical applications often requires substantial computational resources (6, 7, 12), limiting accessibility for multiple iterations during model development. In their work, Doron et al. (8) showed that DINO features could predict expert-defined cellular phenotypes, enhance the prediction of compound bioactivity, and facilitate unbiased profiling of cellular morphology. However, this study also revealed that ImageNet features *can* generalize in some settings more effectively than fine-tuned models in the cellular domain, especially in (rather) low-data regimes. Generally, foundation models, characterized by their vast scale and versatility, are pre-trained on a variety of abstract objectives, enabling them to capture a wide array of features applicable across domains.

DINO leverages self-distillation, allowing the model to *teach* itself by comparing different versions of the same image. SAM focuses on segmentation, learning to identify specific objects within an image based on prompts such as points and bounding boxes. SWIN (13, 14) builds a layered understanding of the image through hierarchical feature maps and directs its attention to specific regions using a *shifted window* approach. ConvNeXT (15, 16) rethinks the traditional convolutional neural network architecture. CLIP (17) learns to associate image content with natural language descriptions. Finally, ViTMAE (18) employs a masked autoencoder technique, hiding parts of the image and tasking the model to reconstruct them. These diverse objectives and architectures enable these models to extract complementary and orthogonal information from the data, potentially leading to better generalization on unseen data outside the training distribution. In line with the *Platonic Representation Hypothesis* (19), we believe this makes them particularly suitable for tasks like cellular imaging analysis, where acquiring large amounts of labeled data can be challenging.

**Figure 1:**
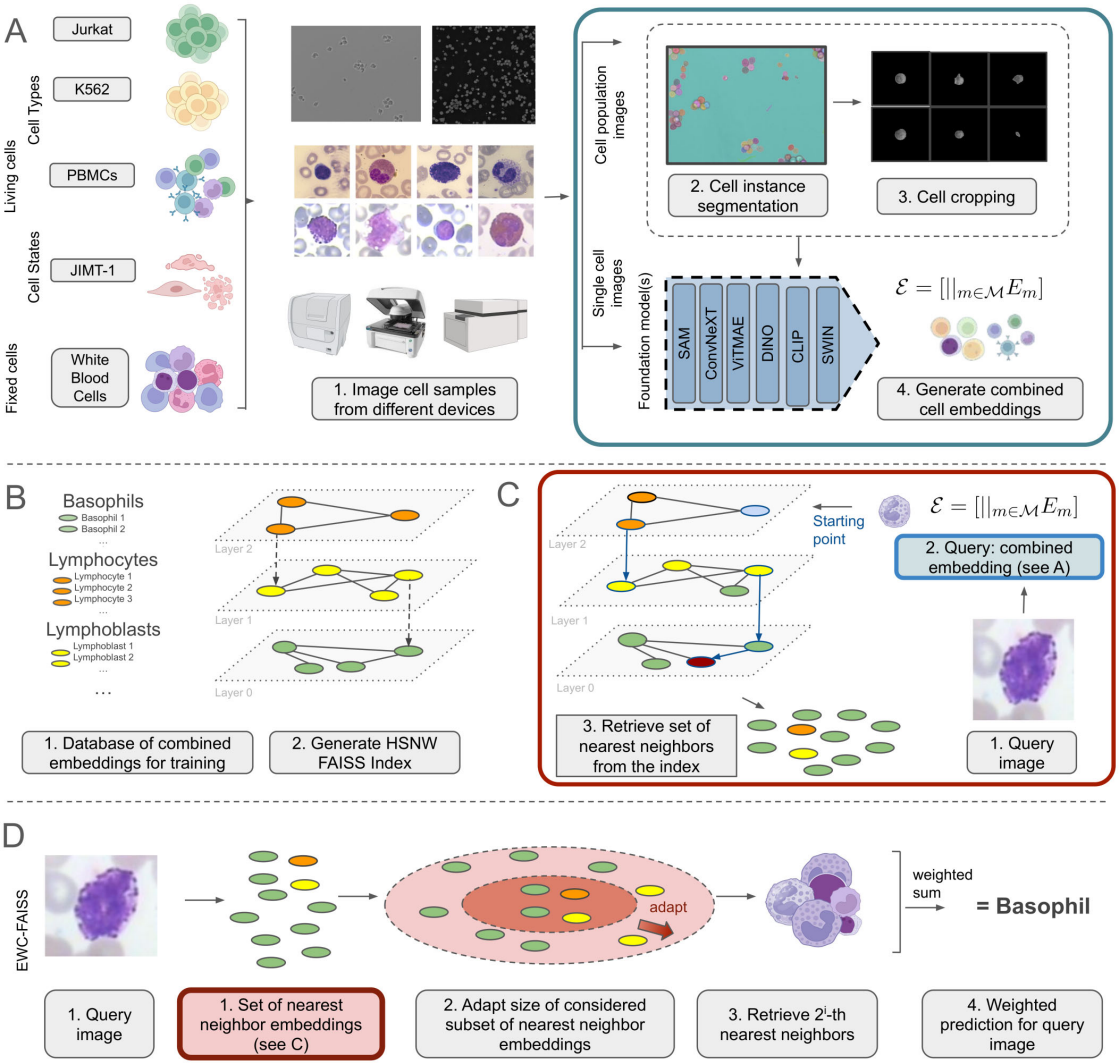
**(A)** Unsupervised cell embedding generation from cell images using a combination of foundation models, with embeddings generated in pure inference-only mode without task-specific fine-tuning. **(B)** Generation of training database and HNSW FAISS index from cell embeddings. **(C)** Retrieving the set of nearest neighbors from the index for a new query image. **(D)** Inference of EWC-FAISS for the prediction of a new image.

**Figure 2:**
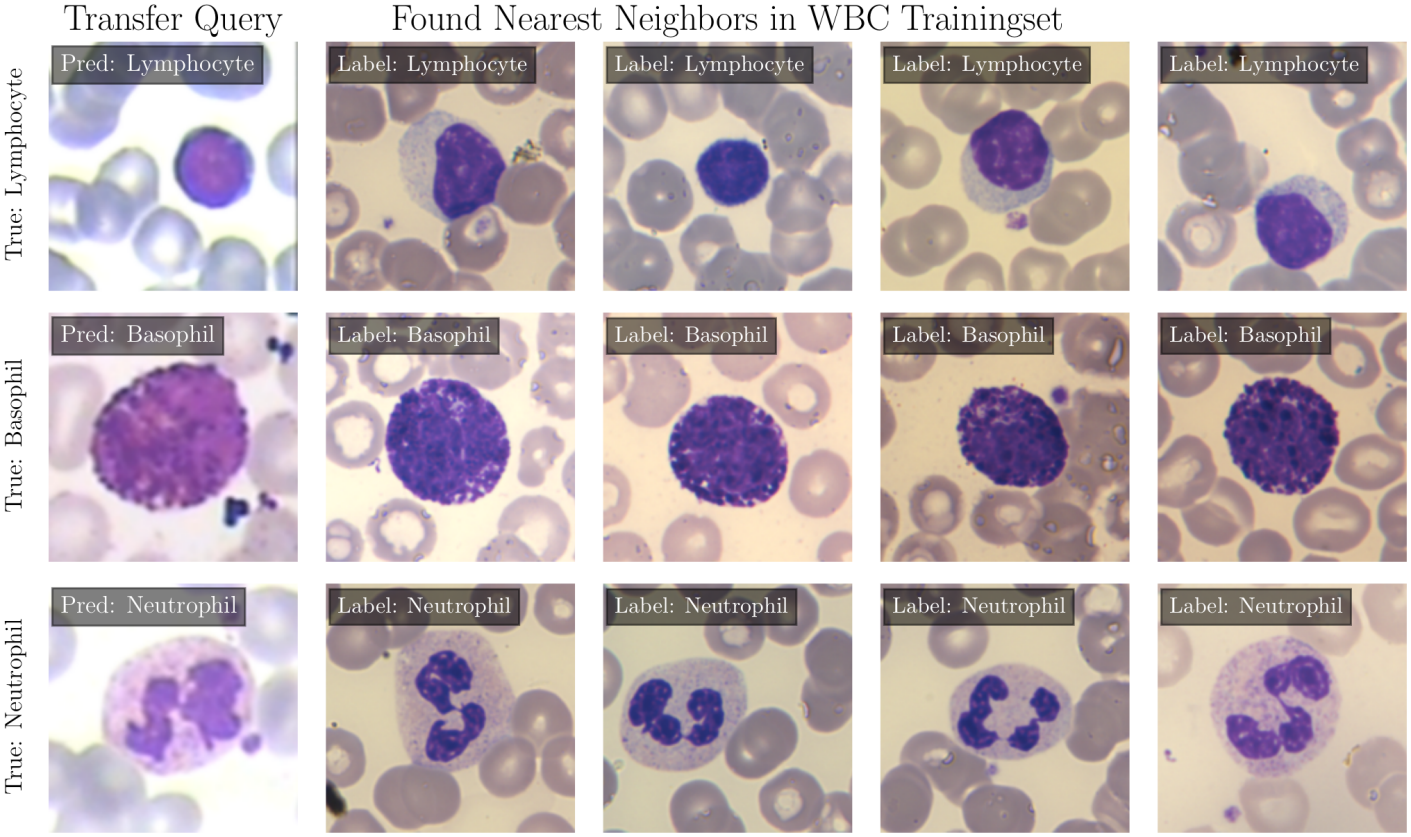
Prediction of EWC-FAISS on query images from the LISC dataset, the four nearest neighbors in the WBC dataset and the ground truth labels.

Following this rationale, this study explores the utility of various foundation models without fine-tuning for the task of cellular imaging analysis. In this context, we developed an automated pipeline, *Entropy-guided Weighted Combinational FAISS* (EWC-FAISS), combining different foundation models as pre-trained feature-extractors to generate concatenated embeddings, which are then used to build an approximate Hierarchical Navigable Small World (HNSW) (20) FAISS index (21) for an efficient k-NN search (see **Figure 1**). To enhance robustness, we propose an entropy-based search for the optimal number of neighbors at runtime, and to alleviate class imbalance through distribution reweighting. Recent research has investigated how to best select foundation models and hyperparameters for cost-efficient fine-tuning for the task at hand (22) and how to make the general features learned from foundation models more robust for downstream tasks via covariance and dominant singular value regularization (23). Our proposed approach stands orthogonal to this line of research by leveraging a combination of features from various foundation models as feature extractors, even when trained on non-domain specific data. This methodology aims to achieve better generalization and adaptability in cellular imaging analysis without any fine-tuning typically required. By building a FAISS index, model iteration can be executed much faster compared to training a full parameterized classifier while still being able to benefit from the generalization capabilities of sophisticated feature extractors (cf. **Figure 2**). We were able to demonstrate, that combining features from multiple foundation models, trained on natural images, can outperform single-model approaches, including DINO, in terms of performance and transferability.

Finally, in this work we successfully validated the effectiveness of our approach in multiple scenarios. First, we performed an evaluation on the WBC dataset (24) containing stained blood cell smears, as well as a transfer to the LISC dataset (25). Second, we conducted an analysis of EWC-FAISS on live cell state classification, with a transfer from the Nanolive CX-A to the BioTek Lionheart FX microscopes. Third, we challenged our approach on live cell type classification, with a transfer from the BioTek Lionheart FX to the Nanolive CX-A. Lastly, we evaluated NMTune (23) in these domains.

## 2 Materials and methods

### 2.1 Data

**Figure 3:**
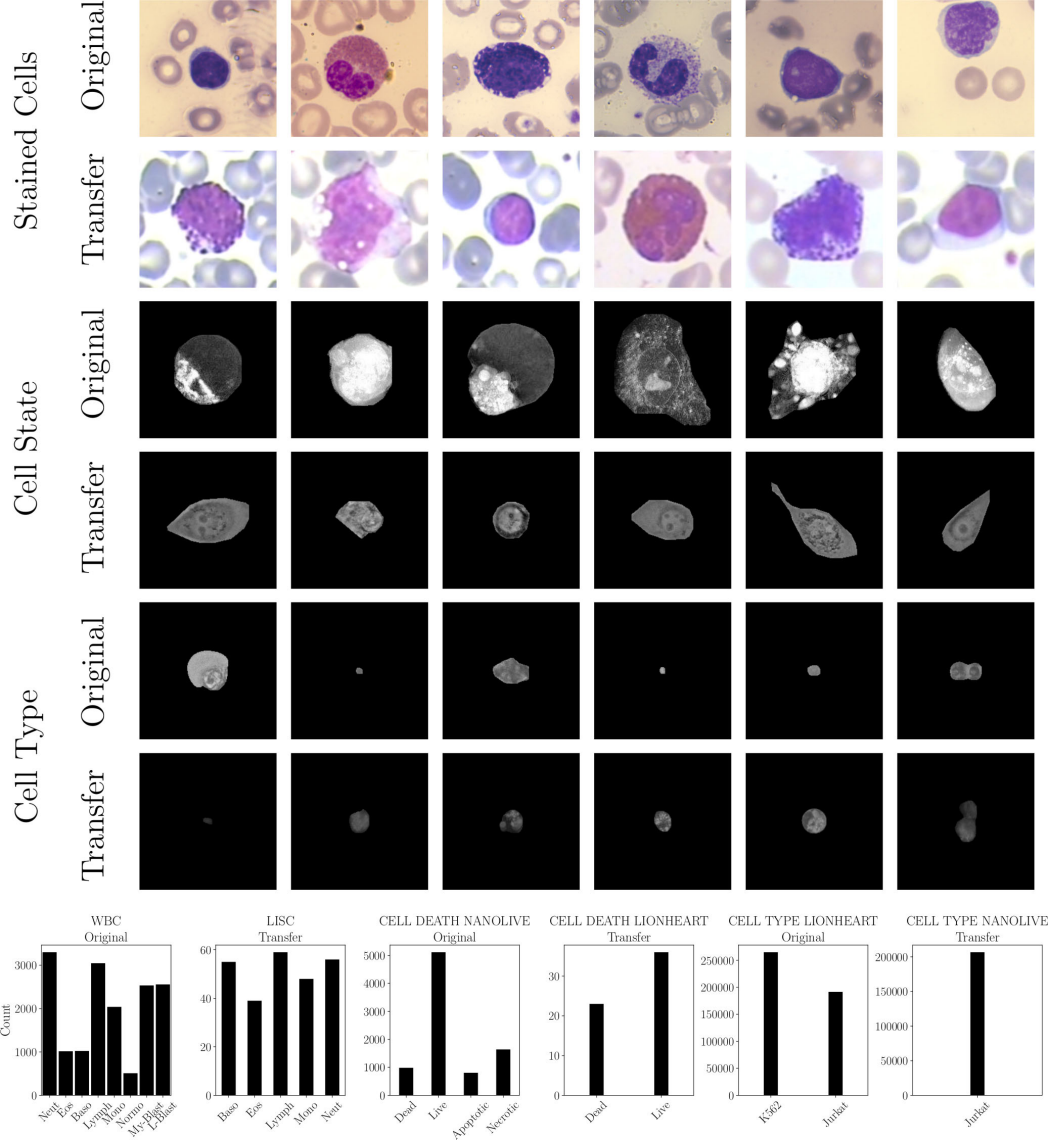
Used datasets: stained white blood cells of the WBC (Original) and LISC dataset (Transfer), live cell state recorded by the Nanolive (Original, CELL DEATH NANOLIVE) and BioTek Lionheart FX microscopes (Transfer, CELL DEATH LIONHEART), and two cell lines recorded by BioTek Lionheart FX (Original, CELL TYPE LIONHEART) and Nanolive (Transfer, CELL TYPE NANOLIVE). (top) Exemplary images. (bottom) Class distributions of all datasets.

We utilized two publicly available datasets, i.e. WBC (24) and LISC (25), and created four new datasets (CELL DEATH NANOLIVE, CELL DEATH LIONHEART, CELL TYPE NANOLIVE and CELL TYPE LIONHEART, cf. **Figure 3**). For all datasets, external and in-house, the labeling was performed by a human domain expert (24, 25). The distribution can be found in **Figure 3**.

#### 2.1.1 Stained white blood cells

The WBC (24) dataset includes 14,424 cell images from microscopic blood smear images from 36 leukemic and 45 non-leukemic peripheral blood smears, collected from 78 anonymized patients. This cohort includes 18 patients with acute myeloid leukemia, 15 with acute lymphoid leukemia, and 45 with no leukemic pathology. Blood smears were stained using May-Grünwald and Giemsa-Romanowski solutions, and blast cell lineage was determined by flow cytometry. Images were captured using an Olympus BX51 brightfield microscope with a Basler acA5472-17uc camera, achieving a resolution of approximately 42 pixels per 1*µm* under a magnification of 100×. The dataset contains nine different annotated blood cell types: neutrophil segments and neutrophil bands (3300), eosinophils (1017), basophils (1023), lymphocytes (3046), monocytes (2040), normoblasts (510), myeloblasts (2534), and lymphoblasts (2557). Due to the low number of neutrophil bands, we have merged them with the neutrophil segments. The LISC (25) dataset contains hematological images from peripheral blood of 8 healthy individuals, resulting in 257 white blood cell images from 100 microscope slides. These slides were stained using the GismoRight technique, imaged with a Zeiss Axioskop 40 brightfield microscope at 100× magnification, and recorded by a Sony SSCDC50AP digital camera in BMP format. We converted the images to grayscale. Each image was collected from the Hematology-Oncology and BMT Research Center at Imam Khomeini Hospital, Tehran. A hematologist classified the images into five normal leukocyte categories: basophil (55), eosinophil (39), lymphocyte (59), monocyte (48), and neutrophil (56).

#### 2.1.2 Live cell state imaging

**Figure 4:**
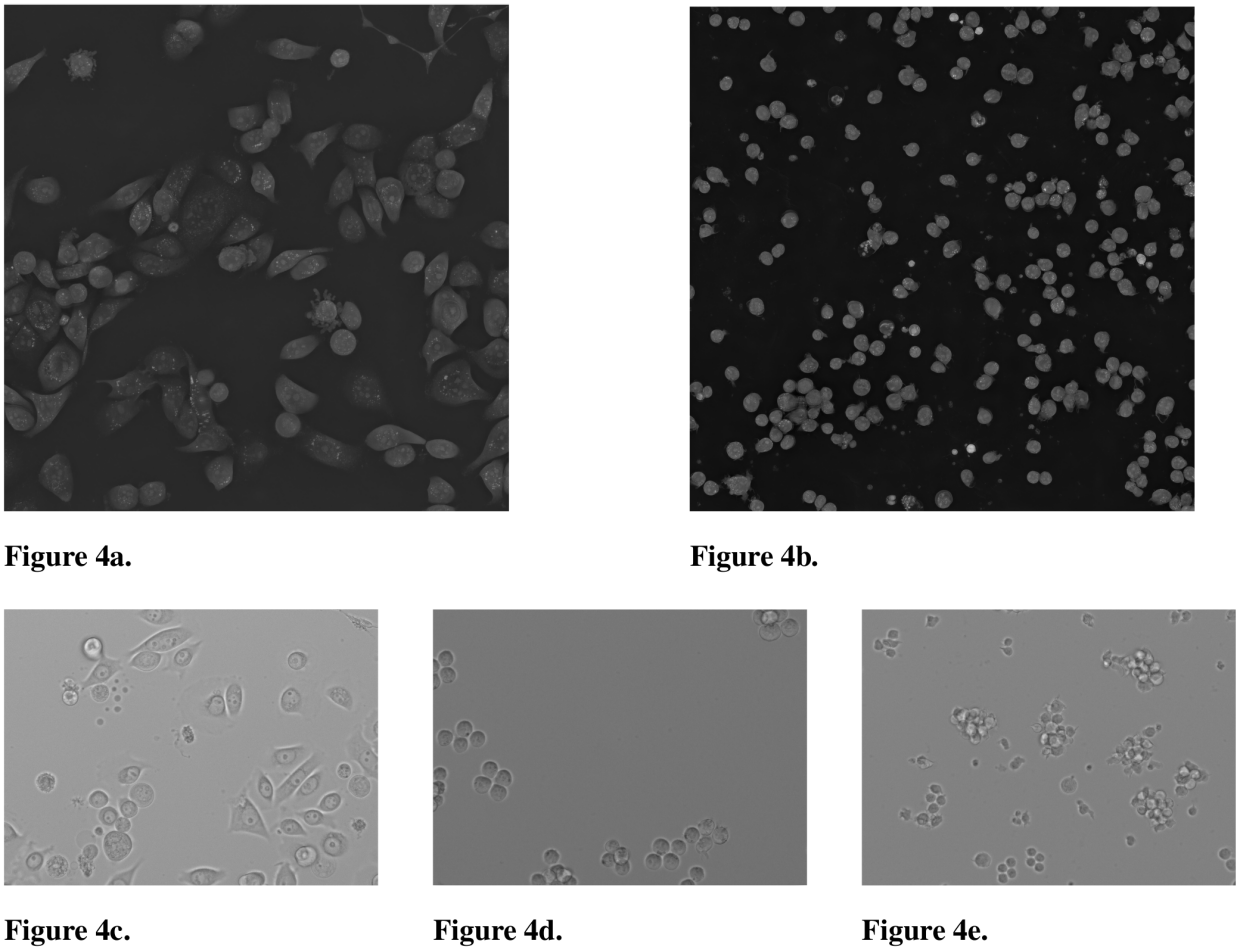
2D maximum intensity projection of the 3D holotomographic Nanolive microscope and images of the brightfield Lionheart microscope showing various cell types. **(a)** CELL DEATH NANOLIVE image of JIMT-1 breast cancer cells. **(b)** CELL TYPE NANOLIVE image of Jurkat cells. **(c)** CELL DEATH LIONHEART image of JIMT-1 cells. **(d)** CELL TYPE LIONHEART image of K562 cells. **(e)** CELL TYPE LIONHEART image of Jurkat cells.

The CELL DEATH NANOLIVE dataset comprises 7,420 images of JIMT-1 cells, captured at 60× magnification using a Nanolive CX-A microscope. The microscope generates several cross sections which are combined to generate a high-resolution 3D holotomographic projection and can be represented as a 2D maximum intensity projection. The dataset is categorized into Dead (728), Living (4,613), Apoptotic (707), and Necrotic (1,372) cells. An additional test set includes 1,122 images, with Dead (255), Living (500), Apoptotic (99), and Necrotic (268) cells. The images are 2D projections of 3D volumes. JIMT-1 cells were cultivated using Dulbecco’s modified eagle medium (DMEM) FluoroBrite (Gibco), supplemented with 10% fetal bovine serum (FBS; Gibco), 1x L-glutamine (Gibco), and 1% Pen Strep (10,000 Units/ml penicillin, 10,000 µg/ml streptomycin; Gibco). JIMT-1 cells were either non treated or treated with 2.5 µM, 5 µM and 10 µM of Raptinal (Sigma Aldrich) for 24h to induce cell death. 300 nM of Biotracker Apo15 (Sigma) was used as a fluorescence marker to detect early stage apoptosis. The cells were seeded in a µ dish 35 mm, low glass bottom (Ibidi). Brightfield Images were acquired every 15 min and fluorescence every 3h. For each image, we used contrast limited adaptive histogram equalization (CLAHE) to normalize its contrast. We used Roboflow for annotations. The CELL DEATH LIONHEART dataset contains 59 annotated test images at 20×, categorized into dead (23) and living cells (36). The breast carcinoma cell line JIMT-1 (ACC 589, DSMZ) was used as adherent cells. JIMT-1 cells were cultivated using Dulbecco’s modified eagle medium (DMEM) (Gibco), supplemented with 10% fetal bovine serum (FBS; Gibco), 1x L-glutamine (Gibco), and 1% Pen Strep (10,000 Units/ml penicillin, 10,000 µg/ml streptomycin; Gibco). Cells were incubated at 37°C in a humidified atmosphere containing 5% CO2 and passaged twice a week. For treatment, JIMT-1 cells were seeded in an 8-well chip (Ibidi) and either left untreated or treated with 25 µM of Etoposide (Sigma Aldrich) for 72 hours. Propidium Iodide (0.25 µg/ml, Sigma Aldrich) was used as a fluorescent marker to stain dead cells. Brightfield and fluorescence images were acquired every 2 hours using a BioTek Lionheart FX automated brightfield microscope. Examples can be found in **Figure 4**.

#### 2.1.3 Live cell type imaging

The CELL TYPE LIONHEART dataset includes 456,366 images of homogeneous K562 (264,904) and Jurkat cell images (191,462), captured using a BioTek Lionheart FX automated brightfield microscope at 20× magnification. The CELL TYPE NANOLIVE dataset consists of 206,742 images of Jurkat cells captured with the Nanolive CX-A holotomographic microscope at 60×. Each image was segmented using SAM, and the type of each crop was assigned accordingly. Contrast normalization was applied to each image using CLAHE. Jurkat and K562 cells were cultivated in RPMI 1640 medium (Gibco), supplemented with Advanced RPMI, 4% FBS, 1x L-glutamine, and 1% Pen Strep. All cells were incubated at 37°C in a humidified atmosphere containing 5% CO2 and passaged twice a week. Peripheral Blood Mononuclear Cells or PBMCs were isolated from LRS chambers obtained from healthy donors from the Institut fur¨ Transfusionsmedizin und Gentherapie, Medical Center - University of Freiburg. Contents of the LRS chambers were diluted in Histopaque^®^-1077 (Sigma Aldrich) and centrifuged according to the manufacturer’s protocol. The isolated PBMCs were counted and viability was estimated using Trypan blue exclusion dye using a Neubauer chamber. PBMCs were maintained in RPMI 1640 medium (Gibco) supplemented with 10% FBS, 1x L-glutamine and 1% Pen Strep. PBMCs were seeded in an 18 well chip (Ibidi) either separately or mixed with Jurkat and K562 cell lines at various ratios (25:75, 75:25 and 50:50) and brightfield images were acquired using Biotek Lionheart automated microscope.

Unless single-cell images were already available, cell images were segmented and masked using SAM, where we cropped 224 × 224 pixel crops around the centers of the found cell masks. Examples can be found in **Figure 4**. We split the original datasets to 90% train, 9% validation, and 1% test sets and used the transfer datasets only for evaluation.

#### 2.1.4 Magnification adaption

We adapted the resolution between Lionheart and Nanolive microscopes. The Lionheart microscope, operating at 20× with a field of view of 291x394µm^2^, generates images of 904x1224 pixels, corresponding to approximately 0.322µm/pixel. In contrast, the Nanolive microscope, set at 60× with a field of view of 85x85µm^2^, produces images of 448x448 pixels, resulting in a resolution of about 0.19µm/pixel. To match the resolution of the images from the Nanolive microscope to those from the Lionheart microscope, a scaling factor of 0.59 was applied, calculated by dividing the Nanolive pixel size by the Lionheart pixel size. Hence, the Nanolive images were downscaled to match the pixel size of the Lionheart.

### 2.2 Models

For DINO, we used facebook/dinov2-giant, for ConvNeXT we used facebook/convnextv2-large-22k-224, for SWIN we used microsoft/swinv2-large-patch4window12-192-22k, for CLIP we used openai/clip-vit-large-patch14 and for ViTMAE we used facebook/vit-mae-huge, with their respective AutoImageProcessor from the HuggingFace Transformers Library (26). For SAM, we used samvith4b8939 from the official implementation at https://github.com/facebookresearch/segment-anything.

### 2.3 EWC-FAISS

Next, we outline how to build, train, and query a latent embeddings database using EWC-FAISS.

#### 2.3.1 Foundation model embedding generation

We utilize a set of foundation models as encoders to generate embeddings for our data. Each foundation model $M_{i}$ in the set $\left\{M_{1},M_{2},\ldots ,M_{n}\right\}$ processes the input data *X* to produce a corresponding embedding $E_{i}=M_{i}(X)$ . Fora given encoder subset $M\subseteq \{M_{1},M_{2},\ldots ,M_{n}\}$ , we concatenate the embeddings $E_{i}$ from each encoder $M_{i}\in M$ to form a full feature representation $E=[|{|}_{m\in M}E_{m}]$ . This concatenated embedding E serves as the input for subsequent tasks.

#### 2.3.2 Database and FAISS index construction

**Figure 5:**
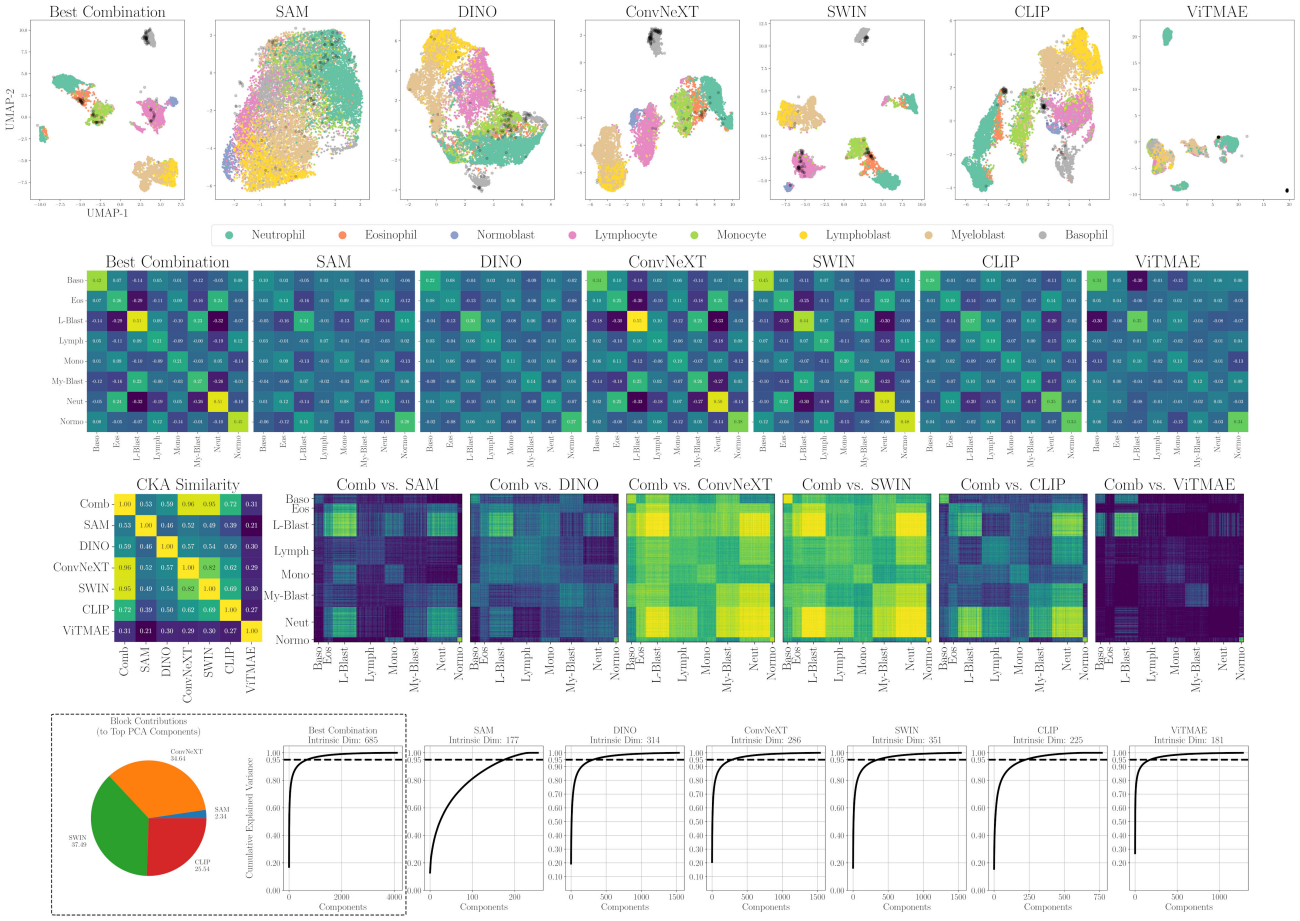
Alignment, similarity and dimensionality analysis of foundation model embeddings for WBC. (first row) 2D projections of individual encoders and SAM+ConvNeXT+SWIN+CLIP (Best Combination). Samples from LISC in black. (second row) Mean cosine similarity scores per cell type or state. (third row) RBF CKA similarity matrix and pairwise comparisons of the combined embedding (Comb) to individual models. Blue indicates low similarity, yellow high similarity. (fourth row) PCA-based intrinsic dimensionality plots, showing the number of components required to explain 95% of the variance, and intra-dataset block contributions of individual encoders to the top PCA components.

**Figure 6:**
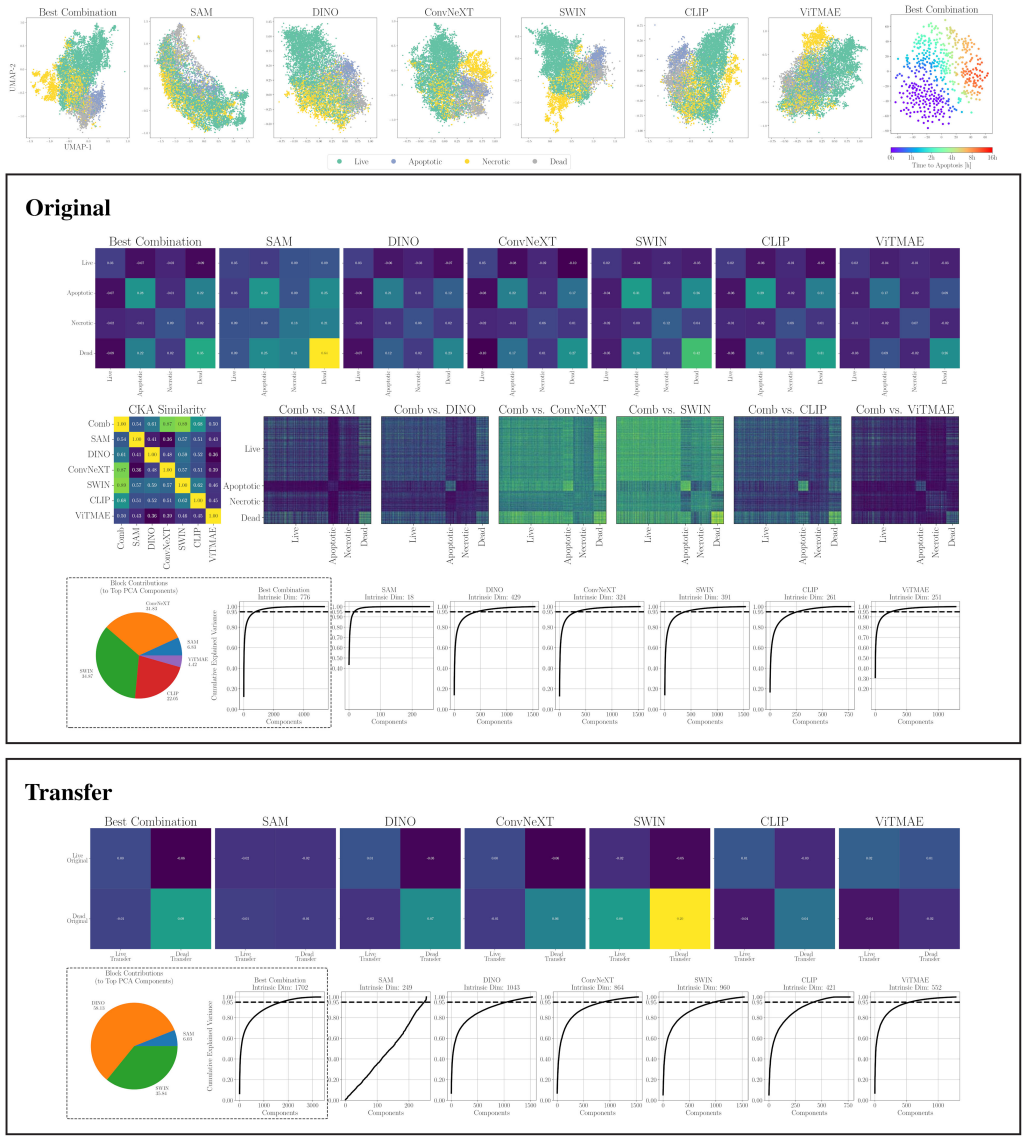
(top) 2D projections of individual encoders and the best combination for CELL DEATH NANOLIVE (left). Embeddings from 15 cells tracked until apoptosis. Color indicates time to apoptosis smoothed by nearest neighbors (right). (middle) Similarity and dimensionality analysis for CELL DEATH NANOLIVE: mean cosine similarity scores (first row); RBF CKA matrix and pairwise comparisons of the combined embedding (Comb) to individual models (second row; blue = low, yellow = high); PCA plots showing components explaining 95% variance and intra-dataset encoder contributions to top components (fourth row). (bottom) Similarity and dimensionality analysis for transfer to CELL DEATH LIONHEART: mean cosine similarity scores (first row); PCA plots and inter-dataset contributions (second row).

We construct a database D consisting of embeddings ${\left\{E_{i}{|}_{i=1}^{n}\right\}}_{c}$ and labels for each cell *c* (cf. **Figures 5**, **6** (top)). This results in $D={\left\{E_{i}{|}_{i=1}^{n}\right\}}_{c=1}^{N}$ , where *N* is the total number of cells. D is then used to train a HNSW FAISS index (20) on concatenated embeddings $E_{c}$ .

#### 2.3.3 Class weight and entropy calculation

To address the issue of class imbalance in our training data, class weights were calculated based on the frequency of each class once after adding the embeddings to the index. The total number of samples was divided by the product of the number of classes and the count of each class. The fixed class weight for class *i* is given by $w_{i}=N/(C\cdot N_{i})$ , where *N* is the total number of samples, *C* is the number of classes, and $N_{i}$ is the number of samples in class *i* in D . This approach ensures that less frequent classes receive higher weights, thereby reducing the impact of imbalance. We then quantify the normalized entropy of labels from the nearest neighbors per sample to estimate the uncertainty in label distribution independent from the re-weighting by Equation 1:


(1) $$H_{\text{norm}}=\frac{-\sum_{i=1}^{m}p_{i}\;log\;(p_{i})}{\log\;(k)},$$

where $p_{i}$ is the probability of the *i*-th class and *k* is the number of nearest neighbors. By normalizing the entropy, it is scaled between 0 and 1 regardless of the number of drawn neighbors. A lower entropy indicates a higher purity of the neighborhood in terms of class labels, which is desirable for accurate classification.

#### 2.3.4 Nearest neighbor search and prediction

The core of our method involves an adaptive nearest neighbor search to determine the optimal number of neighbors (*k*) for classification. Starting from a minimum value $k_{\text{min}}$ , *k* is increased exponentially until the entropy of the nearest neighbors falls below a pre-defined threshold or $k_{\text{max}}$ is reached. This adaptive approach balances the need for accuracy and computational efficiency by dynamically adjusting *k* based on the neighborhood’s label distribution. During the search process, which approximately doubles the runtime per test sample, the class weights are used to perform a weighted vote among the nearest neighbors to account for minority classes. The predicted class is then determined by the class with the highest weighted vote as indicated in Equation 2:


(2) $$y_{\text{pred}}=\text{arg }\underset{l}{\max}\sum_{j=1}^{k}w_{j}\cdot \text{𝟙}\{y_{j}=l\},$$

where $\text{𝟙}\{y_{j}=l\}$ is an indicator function that is 1 if the label of the *j*-th nearest neighbor $y_{j}$ is *l* and $w_{j}$ is the weight of the *j*-th nearest neighbor’s class label.

### 2.4 Experimental setup and hyperparameters

We optimized EWC-FAISS to use the best combination of foundation models (on a validation set) and compared to multiple fine-tuned classification models. Since most related work uses DINO to represent cellular morphology (8, 10), we compared to a (fully-)fine-tuned DINOv2-based vision transformer model (DINO FT), as well as fully-fine-tuned variants of the encoders SWIN and ConvNeXT (SWIN FT and ConvNeXT FT). Furthermore, we compared to a domain-adaption method, NMTune, optimizing a hidden layer following the frozen encoders, as a lightweight addendum to foundation models aiming at making performances more robust on (unseen) downstream tasks.

#### 2.4.1 EWC-FAISS

To create an adaptive and high-performing configuration for EWC-FAISS, we optimized several parameters, including the subset of foundation models used. Given our set of up to six foundation models, we evaluated every possible model combination (all subsets) to find the one that maximized performance. For each subset, we measured macro accuracy on the validation set, selecting the combination with the highest accuracy as the final or *incumbent* configuration. The parameters for $k_{\text{min}}$ , $k_{\text{max}}$ and the entropy threshold were selected empirically based on validation results in preliminary experiments. We did not apply (L2-)normalization or dimensionality reduction, as preliminary validation results also indicated better performance without these steps.

The best-performing index for the WBC dataset used a combination of SAM, ConvNeXT, SWIN, and CLIP with *k* between $k_{\text{min}}=3$ and $k_{\text{max}}=1000$ , an entropy threshold of 0.3, and L2-distance. For the transfer to LISC, we used a combination of DINO, ConvNeXT, SWIN, and ViTMAE. For the cell state classification from the Nanolive microscope, we used a combination of SAM, ConvNeXT, SWIN, CLIP and ViTMAE with *k* between $k_{\text{min}}=3$ and $k_{\text{max}}=100$ , an entropy threshold of 0.6 and Canberra distance. For the transfer to the Lionheart microscope, we set *k* between 10 and 1000 and an entropy threshold of 0.1 with a combination for SAM, DINO and SWIN. For the cell type classification from the Lionheart microscope, as well as for the transfer to the Nanolive microscope, we used a combination of SAM, DINO, ConvNeXT, and CLIP, with *k* between 20 and 1000, an entropy threshold of 0.2 and L2-distance. For the segmentation experiment for real Jurkat and K562 mixtures with PBMCs, we used a combination of SAM, DINO, ConvNeXT, SWIN and CLIP with *k* between 33 and 1000 and an entropy threshold of 0.2 without reweighting.

#### 2.4.2 Fine-tuning baselines

To contextualize our results, we compare against standard fine-tuning approaches using state-of-the-art vision transformers. Specifically, we fine-tune a pre-trained DINOv2 model for 50 epochs using the AdamW optimizer with cosine learning rate decay and warm-up (from an initial learning rate of 10^−5^ to 0). Additionally, we apply the same training setup to SWIN and ConvNeXT backbones. During training, we use horizontal flipping, normalization, and color jitter as data augmentations.

#### 2.4.3 Domain adaption baseline

As a baseline for domain adaptation, we followed a strategy in which the encoder parameters were kept fixed and only an intermediate hidden layer was fine-tuned. Specifically, we employed NMTune, which has been shown to be more robust than conventional layer-wise fine-tuning (23). Following the recommended setup in (23), we set *λ* = 0.01 to balance the regularization terms for feature consistency, covariance alignment, and dominant singular value preservation. We applied NMTune to the optimized embeddings described in Section 2.4.1, as these outperformed the individual encoder embeddings. To reduce computational costs, embeddings were first reduced to 200 principal components using PCA. A two-layer MLP with 800 hidden units was trained for 10 epochs using the Adam optimizer with a learning rate of 10^−3^.

## 3 Results

First, we utilized a set of foundation models as encoders to generate cell sample embeddings as described in Section 2.3.1. Then, we used these embeddings to construct a database and resulting FAISS index as described in Section 2.3.2 to use this as a k-nearest neighbor classifier.

### 3.1 Separation of cell sample embeddings generated from a combination of foundation models

A first intuition into the discriminative capabilities of the general features from foundation models can be seen at the top of **Figure 5**, which depicts a UMAP 2D projection of the latent representations for the different foundation models and the best combination for the WBC dataset. Despite being untrained on domain-specific data, all foundation models except for ViTMAE preserve the local neighborhoods of the different classes, as indicated by the class-specific color coding. When projecting samples from the LISC dataset (i.e., for transfer), which contains only samples from healthy donors, it is evident that ConvNeXT, among all single foundation models, distinguishes best between blasts and non-blasts, with only two miscategorized examples. The best-found combination then has only a single misclassified example according to the 2D visualization. We compared the similarities among cell embeddings within one foundation model exemplary for the WBC dataset in **Figure 5** (second row) by the mean of the cosine similarity scores of all embeddings per cell type projected via Principal Component Analysis (PCA) to 100 dimensions. The best combination of foundation models achieved the highest intra-class similarity with a mean diagonal similarity of 0.355 and the lowest inter-class similarity with a mean off- diagonal similarity of −0.043. In contrast, the ViTMAE model showed the noisiest results, indicating less distinct feature separation. Additionally, we studied the similarity across foundation model representations, using Radial Basis Function (RBF) Centered Kernel Alignment (27) in **Figure 5** (third row). ConvNeXT and SWIN achieved the highest similarity compared to the best combination of models (being part of the best combination SAM+ConvNeXT+SWIN+CLIP). The fourth row of **Figure 5** shows PCA-based intrinsic dimensionality estimates, revealing that the best combination has substantially higher intrinsic dimensionality than any individual model, indicating that it aggregates complementary, non-redundant features across models. To assess how each encoder contributes to the combined embedding, we projected the concatenated representation onto its principal components and quantified the average loading of each encoder’s feature block across the top components, revealing their relative influence on the informative variance. Similarly, a projection of embeddings for cell states from CELL DEATH NANOLIVE can be found at the top left of **Figure 6**. The latent representation from the best combination of foundation models shows the best overall separability between classes, as also depicted in **Figure 6** (middle, first and second row). The third row of **Figure 6** (middle) further shows that this combined embedding retains a higher intrinsic dimensionality, indicating complementary information across models. Lastly, the first row of **Figure 6** (bottom) shows the cosine similarities between states from CELL DEATH NANOLIVE and CELL DEATH LIONHEART. It can be seen, that the different encoders have complementary strengths and weaknesses for separating the single cell states. The found best combination offers a sweet-spot with best overall separation. The bottom row of **Figure 6** (bottom) shows PCA-based estimates of intrinsic dimensionality for embeddings transferred from CELL DEATH NANOLIVE to CELL DEATH LIONHEART. The best combination retains a substantially higher number of informative components, coming from multiple different encoders, than any individual model, suggesting robust generalization.

**Tables 1**, **2** summarize the mean intra-class similarity (average of the diagonal), mean inter-class similarity (average of the off-diagonal), and class separability, defined as the average per class of the difference between the diagonal entry and the sum of off-diagonal entries. These metrics are based on cosine similarity and are reported for all foundation models and their best combination, using embeddings from the WBC and CELL DEATH NANOLIVE datasets.

**Table 1:** Mean intra-class and inter-class similarities, as well as class separability for the WBC dataset.

Model	Intra-class similarity	Inter-class similarity	Class separability
Best Combination	0.355	–0.043	**0.654**
SWIN	0.349	–0.041	0.637
ConvNeXT	0.33	–0.04	0.612
CLIP	0.244	–0.027	0.431
ViTMAE	0.179	–0.021	0.329
DINO	0.183	–0.014	0.283
SAM	0.144	–0.019	0.274

All metrics are based on cosine similarity. *Class Separability* is defined as the average per class of the difference between the diagonal entry and the sum of off-diagonal entries.Values in bold indicate best separability.

**Table 2:** Mean intra-class and inter-class similarities, as well as class separability for different cell states in the CELL DEATH NANOLIVE dataset.

Model	Intra-class similarity	Inter-class similarity	Class separability	Class separability (Transfer)
Best Combination	0.188	0.009	**0**.**162**	**0**.**0772**
ConvNeXT	0.147	-0.004	0.159	0.0626
CLIP	0.176	0.007	0.154	0.0596
ViTMAE	0.130	-0.007	0.152	0.0168
DINO	0.140	-0.001	0.143	0.0769
SWIN	0.215	0.032	0.120	0.0736
SAM	0.290	0.128	-0.093	-0.0003

All metrics are based on cosine similarity. *Class Separability* is defined as the average per class of the difference between the diagonal entry and the sum of off-diagonal entries.Values in bold indicate best separability.

Finally, we present a 2D projection of latent representations from the trajectories of 15 cells taken from the CELL DEATH NANOLIVE dataset and tracked over time by a human domain expert until apoptosis in **Figure 6** (top right). The color indicates the time to apoptosis, smoothed across the respective nearest neighbors. While this does not constitute definitive proof of correct alignment, it illustrates the general tendency of foundation models to capture even subtle morphological differences related to cell state. We further investigate this hypothesis by evaluating classification performance on cell states in Section 3.2.

### 3.2 EWC-FAISS classification performance compared to fine-tuned methods on the original dataset and on transfer datasets

An overview of the classification results over five runs is given in **Table 3**. In terms of within-dataset performance (i.e., trained and tested on the same dataset), EWC-FAISS performs on par or better than the fine-tuned baselines: it achieves the highest accuracy on WBC (97.6%), ties for best on CELL DEATH Nanolive (90%), and performs competitively on CELL TYPE Lionheart (87%). In the transfer setting, EWC-FAISS demonstrates clearly superior generalization. It outperforms all fine-tuned and domain-adapted models by large margins on LISC (78.5% vs. 52% for the next best), CELL DEATH Lionheart (86% vs. 81%), and CELL TYPE Nanolive (85% vs. 63%). Notably, models like DINO FT and SWIN FT degrade significantly in these transfer scenarios, especially on LISC, where DINO FT drops to 17% and SWIN FT to 44%. NMTune, while more than the fine-tuned models, still trails behind EWC-FAISS across all transfer tasks.

**Table 3:** Classification results for models with fully fine-tuned feature extraction (FE), domain adaptation using a frozen backbone (NMTune), and EWC-FAISS with unsupervised FE.

Feature extraction	Method	Macro accuracy	Macro precision
Original: WBC (8 classes)			
	DINO FT	94.0 ± 0.9	98 ± 2
fine-tuned	SWIN FT	92.9 ± 0.8	99.4 ± 0.1
	ConvNeXT FT	92.7 ± 0.5	98 ± 1
domain-adapted	NMTune	98.4 ± 0.5	98.1 ± 0.9
unsupervised	EWC-FAISS	97.6 ± 0.2	98 ± 0
Transfer: LISC (5 classes)			
	DINO FT	17 ± 1	33 ± 2
fine-tuned	SWIN FT	44 ± 14	35 ± 3
	ConvNeXT FT	45 ± 9	49 ± 8
domain-adapted	NMTune	52 ± 10	59 ± 6
unsupervised	EWC-FAISS	78.5 ± 0.3	81.9 ± 0.5
Original: Cell Death Nanolive (4 classes)			
	DINO FT	89.5 ± 0.9	89 ± 1
fine-tuned	SWIN FT	90 ± 4	91 ± 2
	ConvNeXT FT	89 ± 2	91 ± 2
domain-adapted	NMTune	88.4 ± 0.6	89 ± 1
unsupervised	EWC-FAISS	90 ± 0	92 ± 0
Transfer: Cell Death Lionheart (2 classes)			
	DINO FT	65 ± 11	80 ± 21
fine-tuned	SWIN FT	68 ± 16	75 ± 14
	ConvNeXT FT	81 ± 1	85 ± 2
domain-adapted	NMTune	81 ± 7	80 ± 6
unsupervised	EWC-FAISS	86 ± 1	84.9 ± 0.8
Original: Cell Type Lionheart (2 classes)			
	DINO FT	91.9 ± 0.1	92.0 ± 0.1
fine-tuned	SWIN FT	90.6 ± 0.2	90.7 ± 0.2
	ConvNeXT FT	92.32 ± 0.08	92.33 ± 0.05
domain-adapted	NMTune	92.1 ± 0.2	91.9 ± 0.1
unsupervised	EWC-FAISS	87 ± 0	87 ± 0
Transfer: Cell Type Nanolive (1 class)			
	DINO FT	24.5 ± 0.9	–
fine-tuned	SWIN FT	57 ± 6	–
	ConvNeXT FT	54 ± 4	–
domain-adapted	NMTune	63 ± 4	–
unsupervised	EWC-FAISS	85 ± 0	–

Models were trained on the ORIGINAL set and evaluated on both the test and TRANSFER sets. Results are reported over five runs as mean ± standard deviation, rounded to one significant digit.

**Figure 7:**
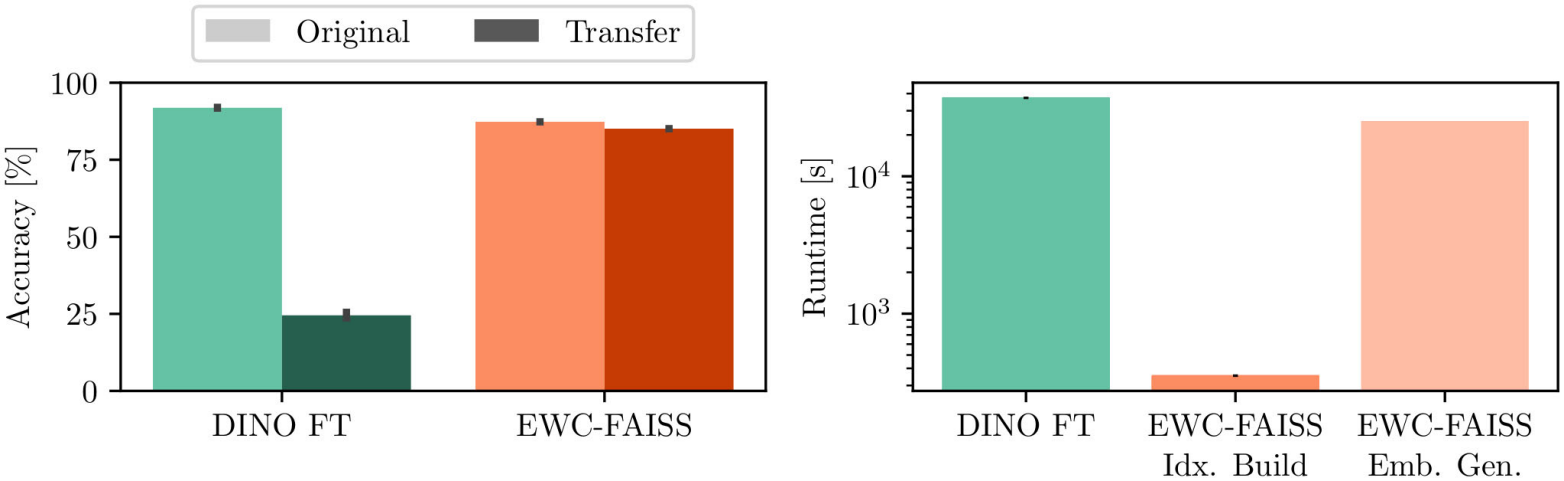
Results on the transfer from a BioTek Lionheart FX to the Nanolive 3D Cell Explorer. (left) EWC-FAISS is robust to distribution shift induced by the second device compared to DINO FT. (right) Once the embeddings are generated (Emb. Gen.), EWC-FAISS is multiple orders of magnitude faster (Idx. Build).

**Figure 8:**
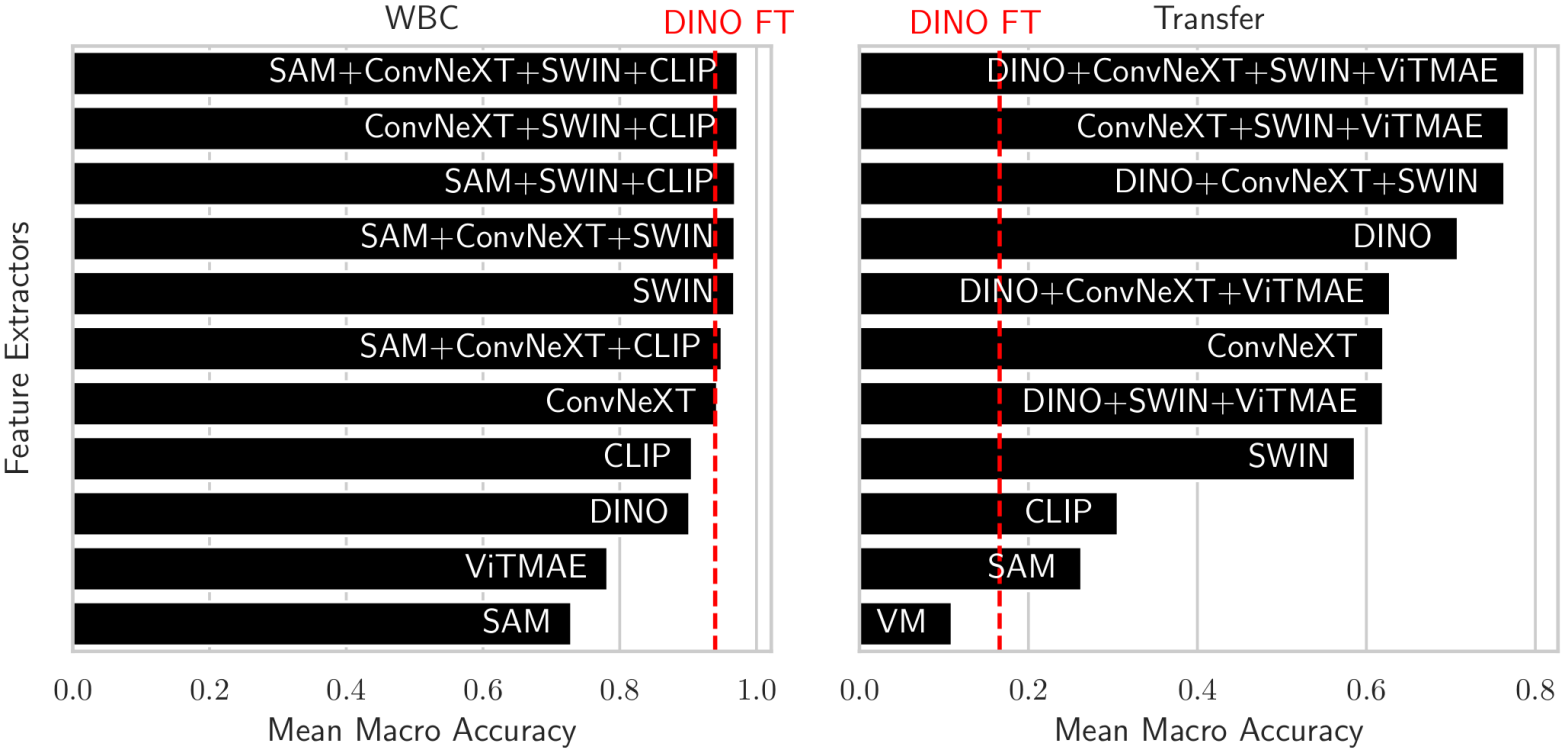
Balanced accuracies of the foundation models and the best combination using EWC-FAISS in black and DINO FT in red on WBC (left) and on the transfer to LISC (right).

**Figure 9:**
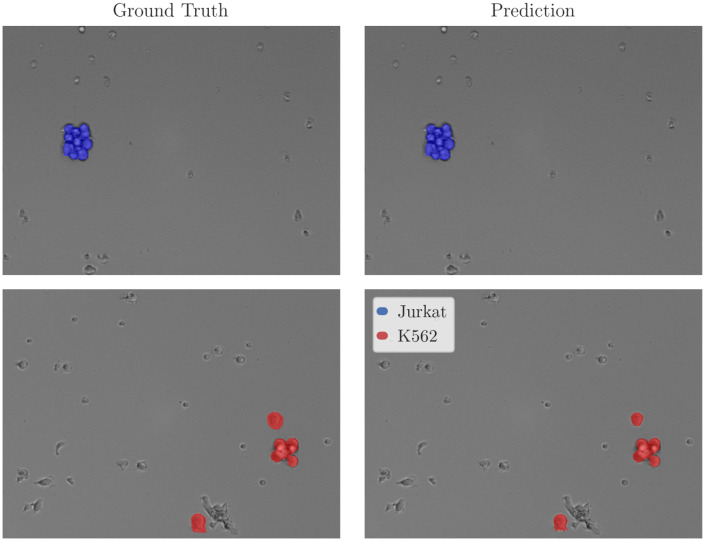
Exemplary inference results for EWC-FAISS evaluated on real mixtures of PBMCs with Jurkats and PBMCs and K562s. Expert annotations were non-exhaustively given for Jurkats and K562s, PBMCs are not annotated.

In addition to performance, EWC-FAISS offers substantial computational advantages. Once the embeddings are computed, building EWC-FAISS is multiple orders of magnitudes faster than training DINO FT (∼ 6 minutes compared to *>* 10 hours, cf. **Figures 7**, **8**) . Lastly, we created real mixtures of Jurkat and K562 cell lines with PBMCs from healthy donors. Our results demonstrate that EWC-FAISS performs comparably to (semi-)supervised segmentation methods (28) in identifying cancerous cell lines within the collection of healthy PBMCs (cf. **Table 4**; **Figure 9**).

**Table 4:** Results of EWC-FAISS on mixtures of PBMCs and Jurkats and PBMCs and K562s.

Method	PBMC+Jurkats	PBMC+K562s	∅
	mAcc	mAcc	mAcc
CellMixer (28)	95.8	63.7	79.7
EWC-FAISS	85.3 ± 0.7	75.2 ± 0.5	80 ± 5

Results are reported as mean ± SD (denoted with one significant digit).

CellMixer results as reported in (28).

### 3.3 Application demonstration: classification of fixed cells

**Figure 10:**
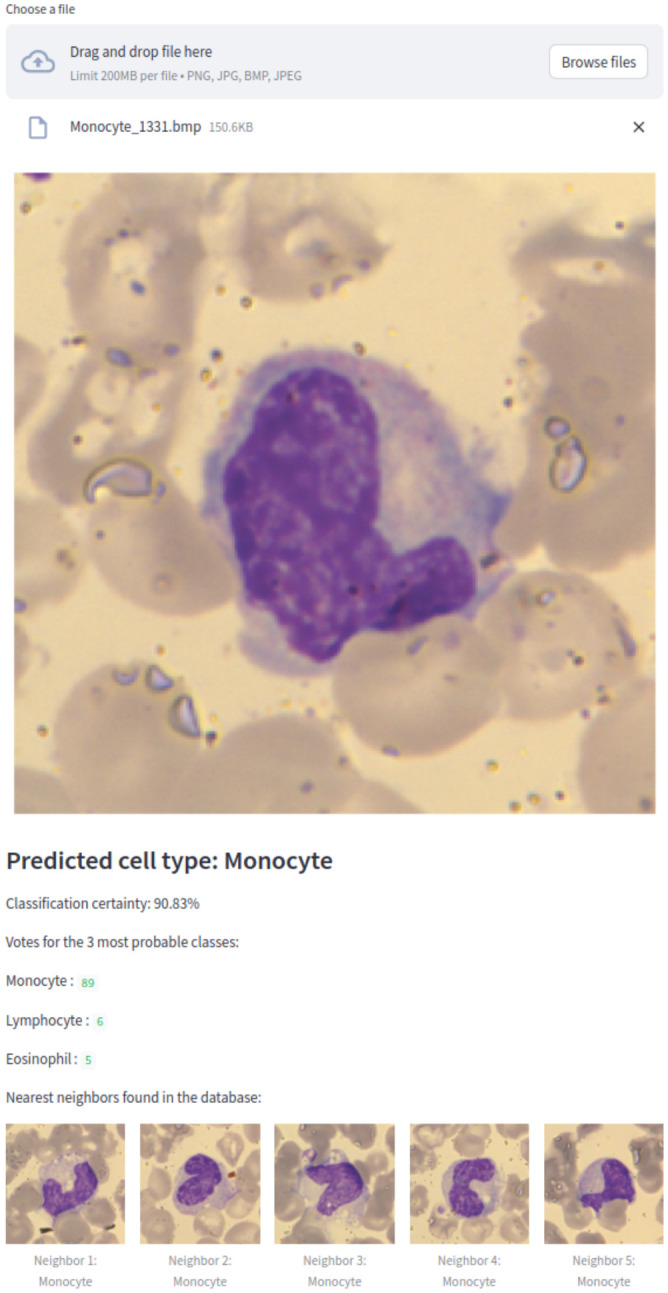
WBC EWC-FAISS classifier within a Streamlit web application predicting a monocyte correctly, showing the classification certainty and the nearest neighbors found in the database.


We implemented EWC-FAISS as a web application to provide a subtype detection for white blood cells using our model trained on WBC. The streamlit^1^ application, shown in **Figure 10** allows to upload an image and shows the prediction together with a certainty measure and the m=5 nearest neighbors of the training set. As measure of certainty, the normalized entropy $H_{\text{norm}}$ was calculated from the label counts of the *k* nearest neighbors. This entropy was normalized relative to the number of nearest neighbors to produce a certainty percentage as an intuitive measure of classification confidence:

$$C=(1-H_{\text{norm}})\times 100\%$$

This application serves as an initial step toward practical clinical use, with the potential to be integrated with a microscope for real-time WBC subtype classification in clinical diagnostics.

### 3.4 Application demonstration: longitudinal classification of cell type and state during live cell analysis

**Figure 11:**
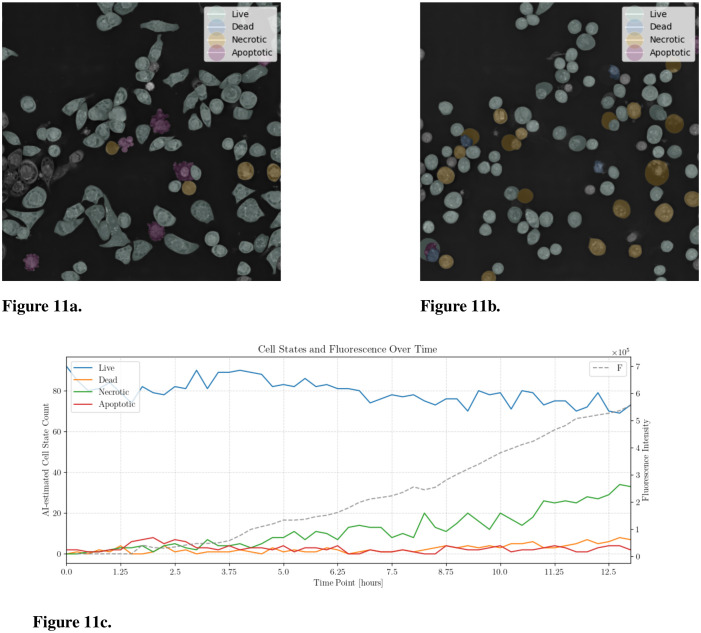
AI-Detection of JIMT-1 cell states using EWC-FAISS trained on NANOLIVE CELL DEATH. The cells were treated with Raptinal (10 µM). **(a)** Timepoint 1.75 hours. **(b)** Timepoint 9.25 hours. **(c)** AI-assisted cell state counts over 12 hours and the fluorescence intensity.

The subtype detection classifier trained on the CELL DEATH NANOLIVE dataset was used to classify the cell state for all images at each time point $t=1,\ldots ,t_{e}$ , where $t_{e}$ is the final time frame of the experiment. Additionally, as a control signal, we calculate the fluorescence intensity by thresholding each Apo-15 fluorescence image using the Otsu method, which determines the optimal threshold by maximizing inter-class variance. A binary mask was created from the thresholded image to highlight regions of interest. These regions were then labeled, and the total fluorescence intensity was quantified by summing the area of the labeled regions. This analysis provides a quantitative measure of the cellular response over the course of the experiment. The analysis of a live cell experiment for JIMT-1 cells treated with 10 µM Raptinal is shown in **Figure 11**.

## 4 Discussion

Using AI-based approaches to optimize cancer therapy, in general, is gaining momentum (29–31). However, distinguishing between different cell types or states from microscopic images, especially in a heterogeneous sample population, still pose significant challenges. Importantly, our results provide evidence that adaptive k-NN search on fixed features from combinations of foundation models (cf. **Figure 8**) can yield matching or better performances in the domain of cellular imaging to distinguish between different cell types or cell states. However, combining features from foundation models also is no panacea and except for the WBC dataset, we had to adapt the combinations and hyperparameters when transferring to a new device. Nonetheless, we were always able to find a good working combination in the realm of minutes instead of hours of training fully fine-tuned models, even for full iterations over the whole power set. This highlights the versatility and accessibility of the proposed framework, as performing several development cycles is very cost-effective and fast; all experiments have been executed on consumer hardware, specifically an AMD Ryzen 9 7950X3 CPU and an NVIDIA GeForce RTX 4090. NMTune on the best found set of foundation models also is a well-performing alternative to a fully-trained classification model, although the generalization capabilities toward out-of-distribution samples appear to be inferior compared to approximate k-NN search. While an exhaustive search over the power set is reasonably cost-effective for smaller sets of foundation models, optimizing the best combination may be challenging in resource-constrained environments or with larger candidate sets. In such cases, the combination of DINO, ConvNeXT, and SWIN has demonstrated consistent performance, particularly for transfer tasks across all evaluated datasets.

The adaptive similarity search on general-purpose features from foundation models proved robust across various microscopy scenarios and devices. This robustness is further demonstrated by achieving not only the highest transfer performance in terms of mean macro-accuracy but also the lowest associated standard deviations. In the LISC transfer task, EWC-FAISS shows exceptional stability with a standard deviation of only ±0.3, compared to NMTune’s ±10, SWIN FT’s ±14 or ConvNeXT FT’s ±9, and less than half of Dino FT’s ±1. For transfer of cell state detection, EWC-FAISS and ConvNeXT FT achieve a variance up to an order of magnitude smaller than Dino FT or SWIN FT (± 1 vs. ± 11 and ±16) and notably lower than NMTune’s ±7. Finally, in the cell type detection transfer task, EWC-FAISS shows negligible variance, compared to NMTune’s ±4, underscoring its consistent performance across various transfer tasks. Finding robust representations of cells is crucial for AI-assisted analysis of the effects of cancer therapies. This robustness is particularly important given the common challenges in medical research, such as small sample sizes and limited data availability. The ability to accurately classify different cell types and tracking changes in real-time using AI-based methods is essential for assessing and accelerating therapeutic impacts. In our study, we successfully distinguished multiple cell types, including leukemic cell lines and PBMCs. This capability is significant, as PBMCs from healthy donors can serve as a model for the tumor microenvironment, providing a heterogeneous mixture of cells similar to patient tumor samples. This aspect of our study underscores the potential application of our method in clinical settings, where differentiating malignant cells from normal immune cells can provide insights into immune infiltration and tumor biology. Furthermore, our methodology demonstrates the capability to identify cell states such as live, apoptotic, necrotic, or dead cells, which for example is vital for evaluating the efficacy of cancer therapies. This functionality is particularly relevant in the context of cancer therapy. Our cell similarity search approach could potentially streamline this process, facilitating the identification of donor cells with strong anti-tumor activity and thus optimizing the preparation and effectiveness of immunotherapies.

In our study, we validated this approach across diverse datasets encompassing various staining techniques and imaging platforms, from conventional microscopy to advanced holotomography-based imaging. The consistent performance across these different datasets highlights our method’s robustness, particularly in clinical contexts involving complex, heterogeneous cell populations. This robustness also suggests potential beyond oncology, where our method’s ability to distinguish molecular characteristics in stained cells could accelerate cytological investigations, helping to identify clinically relevant features in settings such as point-of-care diagnostics. For example, a stained blood sample could be imaged with a basic microscope, uploaded via a web application, and analyzed in real time, providing rapid diagnostic insights. While our study primarily utilized optical and hologram-based microscopy, our approach could generalize effectively to other high-resolution imaging methods. Techniques, which capture high-contrast, visually distinct features, align well with our approach. The data efficiency of EWC-FAISS further supports adaptability, even in domains with limited image throughput.

Going forward, the rapid evolution of foundation models presents a valuable opportunity to enhance our framework. With EWC-FAISS, foundation models can be seamlessly replaced as new models emerge: embeddings can be generated from updated models, and the index rebuilt without altering the underlying workflow. Models such as CellSAM, which have recently emerged, may offer richer feature representations that could further improve the accuracy and robustness of our method. Incorporating more advanced models into our pipeline will likely yield significant benefits, particularly in handling the increasingly complex data generated in modern biomedical research. However, as the diversity of visual foundation models continues to expand, there is a growing need for sophisticated hyperparameter optimization techniques to identify the most effective model combinations.

Lastly, exploring metroid-based solutions offers a promising direction for future research. These methods could provide more efficient alternatives to the full (approximate) k-NN search currently employed, particularly in scenarios involving even large datasets or limited computational resources. By optimizing the computational efficiency of our approach, we can further lower the barriers to its widespread adoption and application in diagnostics and therapy response monitoring.

## 5 Conclusion

In this study, we presented a robust and versatile method for distinguishing between various cell types and states using adaptive k-NN search on fixed features derived from combinations of foundation models. Our approach demonstrated high adaptability and efficiency across different microscopic imaging devices, highlighting its potential for broad applicability in both research and clinical settings. The method’s ability to rapidly adapt to different datasets and experimental conditions, while maintaining accurate classification performance, underscores its utility in dynamic and resource-constrained environments.

## Data Availability

The raw data supporting the conclusions of this article will be made available by the authors, without undue reservation.

## Footnotes

1
https://streamlit.io/
